# Hematemesis is associated with worse outcomes in upper gastrointestinal bleeding: a retrospective study

**DOI:** 10.1590/acb407125

**Published:** 2025-09-29

**Authors:** Gabriela Caetano Lopes Martins, Carolina Villalba Moya Rodrigues, Lucas Domingos Ribeiro, Karin Romano Posegger, Rafael Leite Pacheco, Leonardo de Mello del Grande, Diego Adão

**Affiliations:** 1 Universidade Federal de São Paulo – Escola Paulista de Medicina – Department of Surgery – São Paulo (SP) – Brazil.

**Keywords:** Gastrointestinal Hemorrhage, Hematemesis, Melena, Endoscopy, Gastrointestinal

## Abstract

**Purpose:**

To evaluate whether hematemesis is associated with increased morbidity and mortality for upper gastrointestinal bleeding.

**Methods:**

A retrospective cohort study was conducted at a quaternary university hospital from January 2022 to September 2024. Adults presenting with upper gastrointestinal bleeding, confirmed by endoscopy, were included. We excluded patients with terminal disease, patients who refused to receive blood products, and trauma. The main outcomes were all-cause mortality, need for orotracheal intubation, emergency blood transfusion, need for re-endoscopy, and length of hospital and intensive care unit (ICU) stays.

**Results:**

A total of 69 patients (65% male, mean age 58 years) were included. Hematemesis was associated with a higher need for emergency blood transfusions (73% vs. 23%; *odds ratio* – OR = 8.82, 95% confidence interval – 95%CI 2.44–31.94, *p* = 0.001), longer hospital (12 vs. 6 days; mean difference – MD = 6.02, 95%CI 2.39–9.64, *p* = 0.001) and ICU stays (7.7 *vs*. 3.2 days; MD = 4.5, 95%CI 1.73–7.26, *p* = 0.002). Data were sparse and imprecise on all-cause mortality, orotracheal intubation, and the need for re-endoscopy.

**Conclusion:**

Hematemesis is associated with higher transfusion requirements and longer hospital and ICU stays. These findings highlight the potential predictive value of hematemesis in acute upper gastrointestinal bleeding.

## Introduction

Upper gastrointestinal bleeding (UGIB) is a common medical emergency worldwide, with a mortality rate ranging from 8 to 10%, resulting in more than 400,000 hospital admissions in the United States of America each year^1^. The most prevalent cause of UGIB remains the peptic ulcer and esophageal varices^2^.

The most common clinical manifestations include hematemesis and melena. In current literature, prognostic scores that assess the hemodynamic impact of UGIB, such as the Glasgow-Blatchford score, are complex and do not consider hematemesis to establish the severity of the clinical presentation^3^. There is no consensus that hematemesis is associated with worse prognoses.

The aim of this study was to assess whether hematemesis is associated to morbidity and mortality in UGIB and could predict them.

## Methods

This is a retrospective cohort study, conducted in the emergency department and intensive care units (ICU) at a university hospital in an urban single-center (Hospital São Paulo, São Paulo, SP, Brazil). Data records from electronic medical records were accessed anonymously and encrypted from January 2022 to September 2024.

The study received approval from the institutional ethics committee with the waiver of the consent form (CAAE number 4.725.448), and it is in accordance with the Declaration of Helsinki and good clinical practice guidelines^4^^,^ 5. The report complies with the Strengthening the Reporting of Observational Studies in Epidemiology (STROBE) Guidelines for reporting observational studies^6^.

### Population

Adults (≥ 18 years old) who presented to the emergency department or ICU with the diagnosis of UGIB were included. UGIB was defined as the presence of hematemesis and/or melena at admission, and confirmed by upper endoscopy, regardless of the hemodynamic status or etiology. Patients were divided into two groups: those with hematemesis (bloody or coffee-ground emesis, with or without melena) or no hematemesis (melena, black stools only). We excluded patients with terminal disease, patients who refused to receive blood products, and trauma.

### Outcomes

Data recorded included demographic and medical notes (clinical, laboratorial, risk scores, endoscopic findings, and treatment received).

The main outcomes assessed were all-cause mortality rate, need for orotracheal intubation (during hospitalization due to shock or for airway protection), emergency blood transfusion (need for any kind of blood components in the acute resuscitative phase), need for re-endoscopy (need for a second endoscopy to review or control the source of the bleeding), length of hospital and ICU stays (in days).

### Statistical analysis

Descriptive statistics were expressed as the mean and standard deviation (SD) or median and interquartile range (IQR) for numerical variables, while categorical variables were expressed in percentage (%). Logistic regression was used to assess categorical response variables with the report of *odds ratio* (OR) and 95% confidence interval (95%CI), with robust standard errors. Numerical response variables were assessed by linear regression with the report of mean difference (MD) and 95% CI, with robust standard errors. All analyses were reported unadjusted and adjusted by baseline Charlson score^7^.

The Charlson score was used for the adjustment because it is a score that englobes disease severity in a multi-dimensional aspect considering the following parameters: age, myocardial infarction, congestive heart failure, peripheral vascular disease, cerebrovascular disease, dementia, chronic pulmonary disease, connective tissue disease, peptic ulcer disease, mild liver disease, diabetes without complications, diabetes with complications, hemiplegia, moderate to severe renal disease, moderate to severe liver disease, solid tumor, leukemia, lymphoma, metastatic solid tumor, and acquired immunodeficiency syndrome.

*p* < 0.05 was considered statistically significant. Statistical analyses were performed with the software STATA version 18.

## Results

A total of 69 patients were admitted to the emergency department or ICU, whose demographic and clinical characteristics are shown in Table 1. No patients met the exclusion criteria.

**Table 1:** Demographic and clinical baseline data.

Demographic and clinical characteristics		Hematemesis n = 52, n (%) mean (SD) or median (IQR)	No hematemesis n = 17, n (%) mean (SD) or median (IQR)	Total N = 69, n (%) mean (SD) or median (IQR)
Sex	Male	35 (67.4)	10 (58.9)	45 (65.2)
	Female	17 (32.6)	7 (41.1)	24 (34.8)
Age (years)	-	57.4 (15.6)	49.2 (12.4)	57.98 (15.8)
Glasgow-Blatchford score	-	11.35 (3.1)	9.3 (3.0)	11.0 (7.0)
Liver disease	-	37 (66.1)	8 (61.5)	45 (65.2)
Child-Pugh score	A	14 (25.0)	5 (38.5)	19 (27.5)
	B	14 (25.0)	3 (23.1)	17 (24.6)
	C	16 (46.4)	12 (92.3)	28 (40.6)
Charlson score	-	4 (3; 7)	6 (4; 7)	5 (3; 7)
Rectal bleeding upon digital exam	-	27 (48.2)	9 (69.2)	36 (52.8)
Shock index at admission	-	0.82 (0.61; 1.0)	0.62 (0.59; 0.69)	0.72 (0.59; 0.97)
Hemoglobin at admission (g/dL)	-	8.2 (2.7)	10.4 (2.9)	8.8 (2.9)
Urea-to-creatinine ratio	-	57.95 (25.6)	77.1 (34.7)	65.4 (45.3)
Endoscopic findings	Esophageal varices	13 (23.2)	5 (38.5)	18 (26.1)
	Hypertensive gastropathy	25 (44.6)	5 (38.5)	30 (43.5)
	Gastric ulcer	13 (23.2)	2 (15.4)	15 (21.7)
	Duodenal ulcer	6 (10.7)	1 (7.7)	7 (10.1)
	Neoplasia	5 (8.9)	2 (15.4)	7 (10.1)
Endoscopic treatment	Endoscopic clipping	7 (12.5)	0 (0.0)	7 (10.1)
	Elastic ligature	19 (33.9)	3 (23.1)	22 (31.9)
	Sclerotherapy	1 (1.8)	0 (0.0)	1 (1.5)
	Injection with adrenaline	9 (16.1)	2 (15.4)	11 (15.9)
Non-endoscopic treatments	Octreotide infusion	23 (41.1)	4 (30.8)	27 (39.1)
	Esophageal balloon	4 (7.1)	0 (0.0)	4 (58.0)
	Embolization	1 (1.8)	0 (0.0)	1 (14.5)
	Surgery	3 (5.4)	1 (7.7)	4 (58.0)

IQR: interquartile range; SD: standard deviation.

Source: Elaborated by the authors.

**Figure 1:**
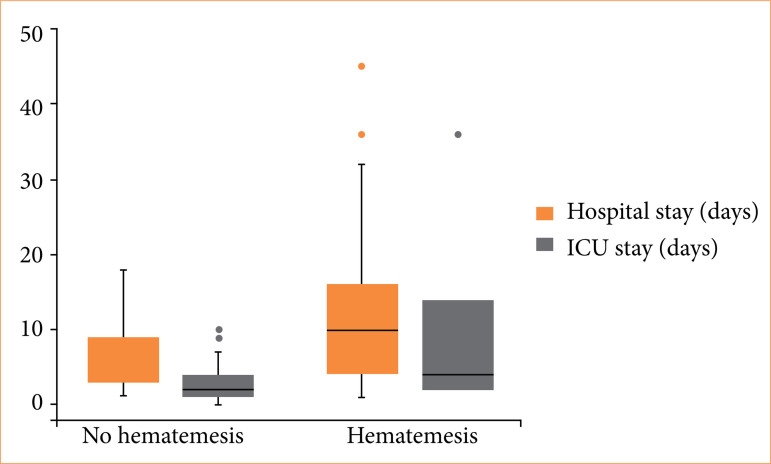
Box plot of hospital and intensive care unit (ICU) stay for patients with and without hematemesis.

Source: Elaborated by the authors.

Patients with hematemesis had 8.8 higher odds (95%CI 2.4–31.94) of need for emergency blood transfusions. Estimates for all-cause mortality, orotracheal intubation, and need for re-endoscopy were imprecise, and confidence intervals were compatible with important outcome odds decrease or increase (Table 2). Furthermore, the presence of hematemesis was associated with a mean increase of six days (95%CI 2.39–9.64) of hospital stay and 4.5 days (95%CI 1.73–7.26) of ICU stay (Fig. 1 and Table 2).

**Table 2:** Clinical outcomes in hematemesis and no hematemesis groups.

Clinical outcomes	Hematemesis n = 52, n (%) or mean (SD)	No hematemesis n = 17, n (%) or mean (SD)	Unadjusted OR or MD (95%CI)	*p* -value	Adjusted OR or MD (95%CI)*	Adjusted *p* -value*
All-cause mortality	12 (23.1)	3 (17.6)	1.40 (0.34–5.76)	0.641	1.47 (0.36–5.95)	0.593
Orotracheal intubation	11 (21.2)	3 (17.6)	1.25 (0.30–5.20)	0.757	1.26 (0.31–5.13)	0.743
Emergency blood transfusion	38 (73.1)	4 (23.5)	8.82 (2.44–31.94)	0.001	10.68 (2.89–39.42)	< 0.001
Re-endoscopy	15 (28.8)	3 (17.6)	1.89 (0.47–7.62)	0.370	1.87 (0.46–7.56)	0.379
Length of hospital stay	12.13 (9.8)	6.12 (5.0)	6.02 (2.39–9.64)	0.002	6.18 (2.71–9.66)	0.001
Length of Intensive care unit stay	7.67 (8.36)	3.18 (3.12)	4.50 (1.73–7.26)	0.002	4.57 (1.83–7.30)	0.001

* Adjusted analysis considered baseline Charlson score;

95%CI: 95% confidence interval; MD: mean difference; OR: *odds ratio*; SD: standard deviation.

Source: Elaborated by the authors.

## Discussion

This study aimed to evaluate whether the specific presentation of UGIB, particularly hematemesis, could be associated with a poor patient prognosis. Our results suggested that hematemesis is associated with a markedly higher need for blood transfusions and longer hospital and ICU stays. However, data regarding all-cause mortality, orotracheal intubation, and the need for re-endoscopy were imprecise and sparse.

The various manifestations of UGIB concern specialists when predicting patients’ outcomes, despite not being explicitly included in current scoring systems. The Glasgow-Blatchford score, one of the most accurate prognostic scores for upper UGIB, does not include a specific item related to hematemesis, but only melena^3^. A similar approach is observed in other scoring systems, such as the Rockall^8^ and AIMS65 scores^9^. This may lead emergency healthcare professionals to perceive that the mode of hemorrhage presentation does not have significance in determining the prognosis of patients.

A prospective study by Blatchford et al.^10^, which laid the groundwork for the Glasgow-Blatchford score system, identified hematemesis as an independent risk factor for mortality (relative risk – RR = 1.9, 95%CI 1.2–2.9) in a cohort of 1,882 patients. Similarly, Laine et al.^11^ demonstrated that patients presenting with hematemesis, whether as bloody or coffee-ground emesis, had a higher mortality rate compared to those with melena alone. Li et al.^12^ also observed that cirrhotic patients with hematemesis had significantly higher rates of five-day rebleeding (17.4% *vs*. 10.1%, *p* = 0.004) and in-hospital mortality (7.9% *vs*. 2.4%, *p* = 0.001). However, Chaikitamnuaychok and Patumanond^13^ performed a multivariate risk analysis and identified melena, rather than hematemesis, as an independent risk factor for severity (*p* < 0.001), whereas hematemesis showed no significant association (*p* = 0.467). In a similar manner, Laine et al.^11^ found that patients presenting with melena had higher rates of hemostatic intervention compared to those with bloody emesis (21.2% *vs*. 19.4%, OR = 0.66, 95%CI 0.51–0.85) and required more blood transfusions compared to those presenting with coffee-ground emesis (53.8% *vs*. 27.4%, OR = 1.49, 95%CI 1.07–2.07). Therefore, a certain divergence in the literature is noted regarding which clinical presentations predict worse prognoses in UGIB.

Additionally, in our baseline, the no hematemesis group included a higher proportion of patients with advanced liver disease (Child-Pugh C cirrhosis with variceal bleeding) and severe comorbidities (Charlson comorbidity index ≥ 5), which are known to be associated with worse prognosis^14^^,^ 15. This imbalance may have led to an underestimation of the true morbidity and mortality difference between the groups, as we can see in the adjusted analysis.

Our findings added to the existing literature by reinforcing the role of hematemesis as a possible marker of severity in UGIB. These results align with the anatomical and physiological rationale that the volume of the bleeding could be directly associated with worse outcomes, regardless of the source or baseline disease.

Based on our findings, we propose some additional measures for managing patients with or without hematemesis in emergency settings. First, emergency protocols should recognize hematemesis as a significant risk factor in UGIB, warranting prioritization of these patients and the initiation of prompt resuscitation measures. Moreover, future UGIB risk scoring systems should consider incorporating hematemesis as an independent criterion, given its association with worse clinical outcomes. These findings underscore the clinical relevance of hematemesis in the early management of UGIB patients, particularly in terms of resource allocation and the intensity of care required.

Our study has limitations. The small convenience sample size and the predominance of elderly, male patients with liver disease introduce potential sources of imprecision and selection bias, which may limit the generalizability of our findings. Additionally, it was a retrospective study, so medical records may be incomplete. It is also importante to note that patients were categorized into two groups (hematemesis and no hematemesis), without distinction between bloody or coffee-ground emesis. However, as Laine et al.^11^ found no significant difference in severity between these two forms of hematemesis, we believe that analyzing them together as a single group was a valid approach. That said, some individuals in the hematemesis group presented other concurrent bleeding manifestations, such as melena. This could introduce bias, based on the same findings by Laine et al^11^, who reported that outcomes associated with hematemesis worsen when melena is present.

Further prospective studies with larger, more diverse populations are warranted to confirm these results and explore the integration of hematemesis into future risk stratification models.

## Conclusion

This study suggested that hematemesis is associated with an increased need for emergency blood transfusions and longer hospital and ICU stays, underscoring its potential as a predictive marker in UGIB. Early identification of hematemesis could play a critical role in guiding clinical decision-making, prioritizing interventions and resources in the acute phase of UGIB management.

## Data Availability

The data will be available upon request.
